# Genetic Variability and Evolution of Hepatitis E Virus

**DOI:** 10.3390/v11050456

**Published:** 2019-05-18

**Authors:** Putu Prathiwi Primadharsini, Shigeo Nagashima, Hiroaki Okamoto

**Affiliations:** Division of Virology, Department of Infection and Immunity, Jichi Medical University School of Medicine, Tochigi 329-0498, Japan; d1631@jichi.ac.jp (P.P.P.); shigeon@jichi.ac.jp (S.N.)

**Keywords:** hepatitis E virus, genetic variability, genotypes, evolution

## Abstract

Hepatitis E virus (HEV) is a single-stranded positive-sense RNA virus. HEV can cause both acute and chronic hepatitis, with the latter usually occurring in immunocompromised patients. Modes of transmission range from the classic fecal–oral route or zoonotic route, to relatively recently recognized but increasingly common routes, such as via the transfusion of blood products or organ transplantation. Extrahepatic manifestations, such as neurological, kidney and hematological abnormalities, have been documented in some limited cases, typically in patients with immune suppression. HEV has demonstrated extensive genomic diversity and a variety of HEV strains have been identified worldwide from human populations as well as growing numbers of animal species. The genetic variability and constant evolution of HEV contribute to its physiopathogenesis and adaptation to new hosts. This review describes the recent classification of the *Hepeviridae* family, global genotype distribution, clinical significance of HEV genotype and genomic variability and evolution of HEV.

## 1. Introduction

Hepatitis E virus (HEV) is the leading cause of enterically transmitted viral hepatitis worldwide. The infection is generally self-limiting; however, infection in immunocompromised patients can cause chronic hepatitis. Fatal cases of acute fulminant hepatitis have been reported in pregnant women, people with underlying liver disease and elderly people [1,2,3,4]. Besides causing typical hepatitis, the infection can also cause extrahepatic manifestations, such as neurological abnormalities, and kidney failure [5]. Over the past several decades, HEV strains have been isolated increasingly frequently not only from humans but also from a broad range of animal species.

HEV infection is distributed globally in both developing and industrialized countries. There are around 20 million cases worldwide, with approximately 3.3 million symptomatic cases annually. The World Health Organization (WHO) estimated that HEV infection caused approximately 44,000 deaths in 2015 (3.3% of mortalities due to viral hepatitis) [6]. The fatality rate in young adults reached 0.5–3% [3]. HEV infection has a poor prognosis among pregnant woman, especially in the third trimester, where the fatality rate can reach up to 30% [7,8,9]. The route of transmission is highly variable but is dominated by fecal–oral and zoonotic routes, such as through the ingestion of raw or undercooked meat, viscera or dairy products of infected animals and close contact with animals (people engaged in high-risk occupations, including veterinarians, workers at slaughterhouses, animal farmers and workers at markets vending animal products) [10,11]. Other modes of transmission that have been recognized with increasing frequency in industrialized countries include blood transfusion and organ transplantation [12,13,14,15,16]. The virus can also be vertically transmitted from infected mothers to their fetuses, resulting in premature birth and stillbirth [9].

HEV infection has been recognized as a self-limiting acute infection transmitted through the fecal–oral route. However, chronic cases can occur in immunocompromised patients, such as those receiving organ transplants, patients with hematological malignancy and human immunodeficiency virus (HIV)-infected patients [16,17,18]. The clinical manifestations can range from typical acute hepatitis to extrahepatic manifestations. Extrahepatic manifestations caused by HEV infection include: (i) neurological abnormalities, which typically present as Guillain–Barre syndrome, neuralgic amyotrophy, encephalitis and myelitis with common characteristics of a monophasic disease course, subacute onset and rapid progression [2,19]; (ii) kidney injury due to membranoproliferative glomerulonephritis with or without cryoglobulinemia and membranous glomerulonephritis; and (iii) hematological disorders, such as hemolytic anemia due to glucose phosphate dehydrogenase deficiency and thrombocytopenia [5,20]. Less-often-reported extrahepatic manifestations associated with HEV infection include acute pancreatitis and autoimmune diseases, such as myocarditis and thyroiditis [5,20].

## 2. Taxonomy

**Figure 1:**
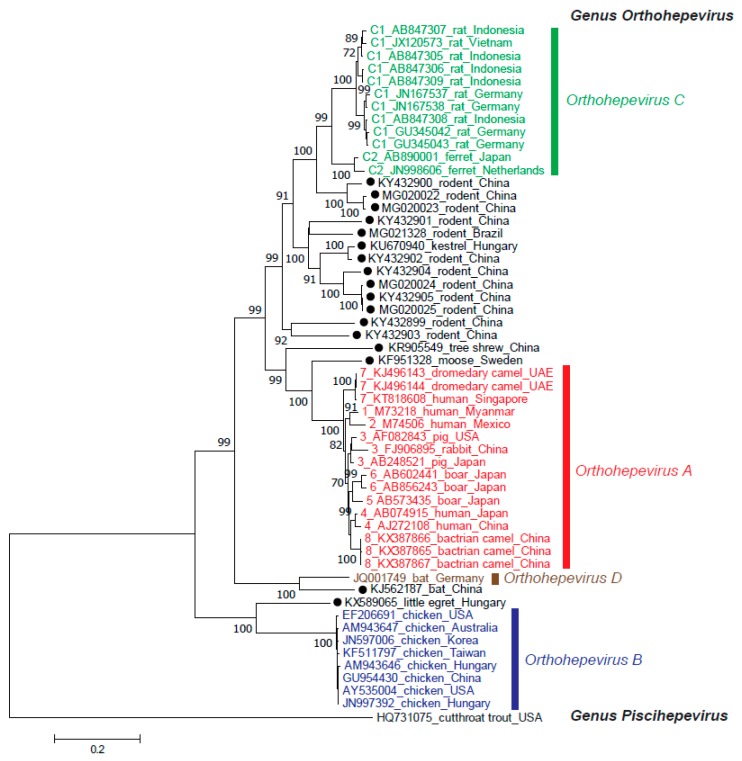
Phylogenetic tree for members of the *Hepeviridae* family. The maximum-likelihood tree was created using MEGA 7 [24] based on amino acid sequences of the entire ORF2 region from members of the *Hepeviridae* family. Each reference sequence is shown with the genotype (if available) followed by the accession number, species (animal/human) and the country where it was isolated. Unassigned HEV strains are highlighted with closed circles. The bootstrap values (>70%) for the nodes are indicated as percentage data obtained from 1000 resampling analyses. The scale bar indicates the number of nucleotide substitutions per site.

Since the discovery of HEV in 1983 [21] and the first report of HEV genomic sequence eight years later [22], the strains classified to the *Hepeviridae* family have been widely identified not only in humans but also in a great number of animal species. With the dramatic increase in the number of HEV strains identified within the past decade, many strains remain unclassified at present, underscoring the marked genomic variability among HEV strains. The recent consensus has divided this family into two genera: genus *Orthohepevirus,* which includes HEV strains from mammals and birds [23]; and genus *Piscihepevirus,* which consists solely of the species *Piscihepevirus A* and its single member, cutthroat trout HEV. Genus *Orthohepevirus* is divided into the four species *Orthohepevirus A, Orthohepevirus B, Orthohepevirus C* and *Orthohepevirus D* [23], which have distinct host ranges (Table 1). Of note, however, many HEV strains—including those isolated from rodents, three shrew species, moose and little egret—remain unassigned, as depicted in Figure 1.

### 2.1. Orthohepevirus A

Species *Orthohepevirus A* has been assigned to eight genotypes (HEV-1 to HEV-8) [23]. The latest addition to this species is HEV-8, which was isolated from Bactrian camels [41]. Species *Orthohepevirus A* has been isolated from mammals―mostly humans, but also pigs and wild boars. This species has also been isolated from a wide range of animals, including deer [42,43], mongooses [44,45], rabbits [46,47,48], dromedary camels [49], yaks [37], sheep [31,35], goats [25,36], horses [26,27,28,29], cattle [32], cows [33,34] and bottlenose dolphins [30] (Table 1).

Although HEV-3 and HEV-4 have been detected in many animal species that were thought to be new animal reservoirs for HEV infection, most can probably be considered: (i) spillover infections related to contact with pigs—the primary reservoir of HEV infection—from the mixed raising of domestic livestock [50]; (ii) spillover hosts rather than a true reservoir, due to the low seroprevalence despite the presence of HEV RNA, as was recently reported in equines in Spain [29] and probably in yaks [37]; or (iii) spillover infection related to the low positivity of HEV RNA despite its high seroprevalence, as was reported in sheep [35]. The reported HEV-3 detected in bottlenose dolphins was also attributed to an environmental contamination with food or wastewater as a source of HEV exposure and infection [30]. Spillover infection may also be responsible for the detection of HEV-4 in dairy milk from cows with high positive rates in Yunnan province in China [34], which has garnered quite a bit of interest. However, this finding is in contrast with those of recent studies in Hebei province of China [51], Germany [52] and Belgium [53]. Those studies found no evidence of HEV prevalence or HEV RNA in milk samples or dairy specimens from cows, suggesting that there is no zoonotic transmission risk through dairy milk from cows to humans.

Despite the serological evidence supporting the existence of an HEV-related agent, extensive attempts to genetically identify HEV-related sequences in cattle from different regions in the USA using broad-spectrum reverse transcription polymerase chain reaction (RT-PCR) assays and MiSeq deep sequencing technology have failed. This suggests the limited ability of this modality for interpreting HEV serological data reported in large numbers of animal species, including cattle. That same study also mentioned that the seroconversion in cattle is caused by antigenic cross-reaction with a related but as yet unknown agent [54]. HEV detected in potential new animal reservoir may also merely be genetically divergent strains of currently recognized HEV strains, as has been reported for goat HEV. Some reports have described the detection of HEV-3 or HEV-4 in goats [25,36]; however, others have suggested that the HEV detected in goats is likely genetically very divergent from the known HEV strains [55]. In the study of Sanford et al. [55], the authors documented serological evidence of HEV infection in the goat population in the USA, but failed to experimentally transmit human or swine HEV to goats; in addition, they were unable to genetically identify HEV from goats using universal degenerate HEV primers based on the sequences of known HEV strains, suggesting that the HEV strain infecting goats is genetically unique [55]. To establish the role of potential new animal reservoirs in HEV transmission and zoonotic disease, the virus should be definitively and reproducibly identified from the animals in question. Therefore, HEV serological data should be carefully interpreted.

Among the four major genotypes of HEV that are capable of infecting humans and belong to species *Orthohepevirus A* (HEV-1 to HEV-4), HEV-1 and HEV-2 are restricted to humans and associated with outbreaks in developing countries where the virus is transmitted through the fecal–oral route, while HEV-3 and HEV-4 are zoonotic with an expanded host range and are the main cause of sporadic and autochthonous cases of hepatitis E in developed countries. Zoonotic cases caused by HEV-3 and HEV-4 strains are mostly associated with strains from pigs and wild boars. However, several reports have noted that HEV-3 strains from other animals are also responsible for causing human infection. The variant of HEV-3 found in rabbits has been isolated from humans in France [56], while HEV-3 from deer was found to cause infection in two families in Japan [42,43]. HEV-5 and HEV-6 have only been isolated from wild boars in Japan [57,58]. HEV-7 was isolated from dromedary camels [49,59], with one report of chronic human infection from the regular consumption of milk and meat of dromedary camels [60], and HEV-8 was isolated from Bactrian camels [41]. HEV-5 and HEV-8 were experimentally transmitted to cynomolgus macaques [61,62,63], demonstrating the possibility of zoonotic infection of HEV-5 and HEV-8.

**Figure 2:**
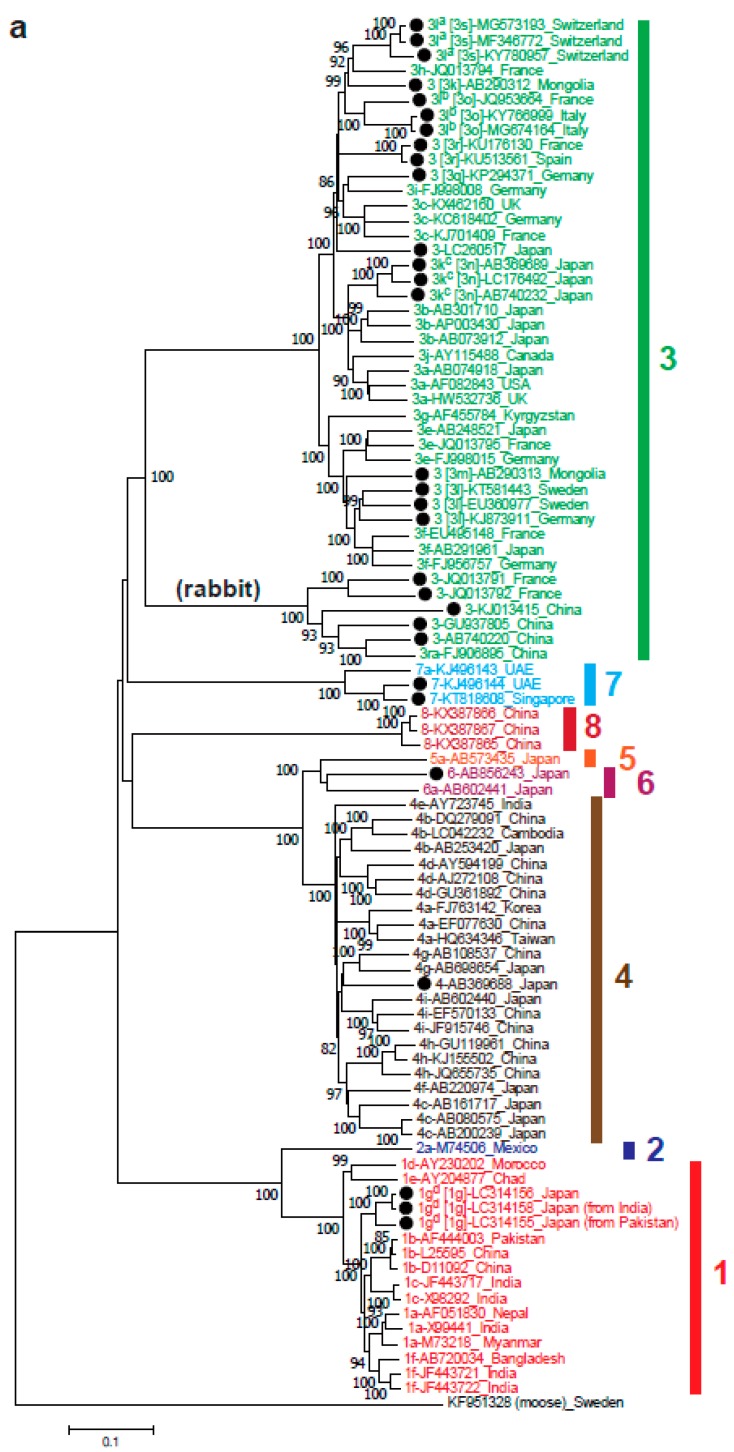

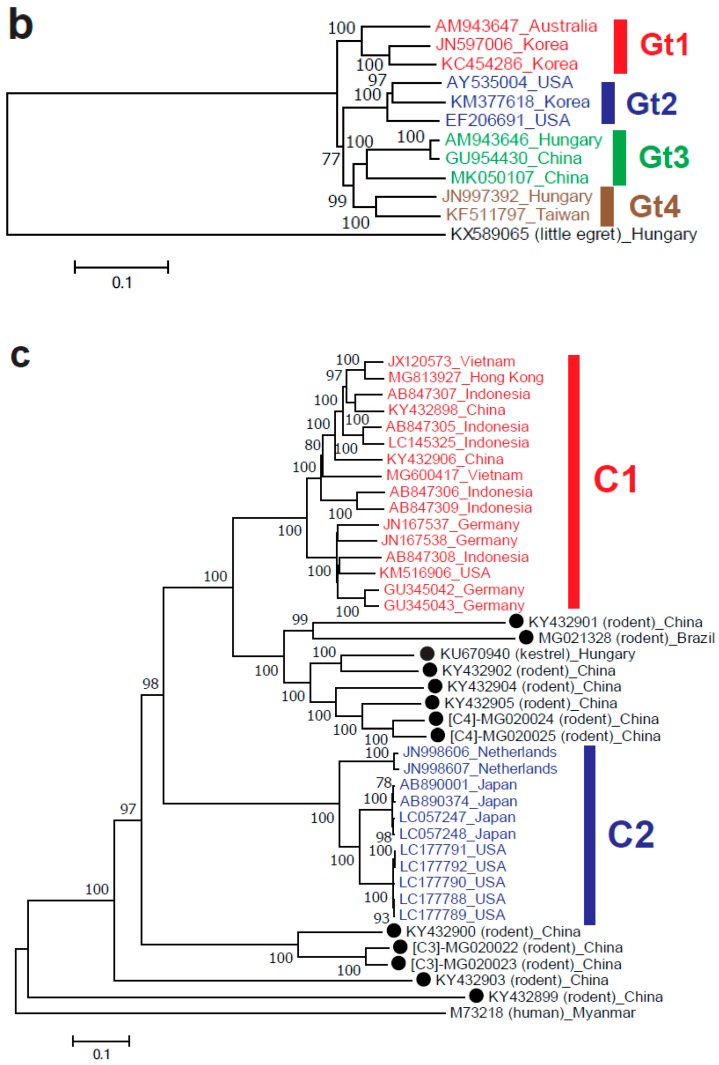
Phylogenetic trees for member of the *Orthohepevirus* genus. Maximum-likelihood trees were created using MEGA 7 [24] based on entire genomic sequences from members of the *Orthohepevirus A* species (**a**), *Orthohepevirus B* species (**b**) and *Orthohepevirus C* species (**c**). Each reference sequence is shown with genotype/subtype (if available) followed by accession number and the country where it was isolated. (**a**) Subtypes 3l^a^, 3l^b^, 3k^c^ and 1g^d^ have been proposed by Wang et al. [68], De Sabato et al. [69], Miura et al. [67] and Nishizawa et al. [65], respectively, and those provisionally proposed by HEVNet (https://www.rivm.nl/en/hevnet) are indicated in brackets. (**b**) Provisional genotypes 1–4 for avian HEV strains are abbreviated as Gt1, Gt2, Gt3 and Gt4, respectively. (**c**) Genotypes C3 and C4 proposed by Wang et al. [78] are indicated in brackets. Reference HEV sequences from moose (KF951328) (**a**), little egret (KX589065) (**b**) and human (HEV-1) (M73218) (**c**) were used as an outgroup. Unassigned HEV strains are highlighted with closed circles. The bootstrap values (>70%) for the nodes are indicated as percentage data obtained from 1000 resampling analyses. The scale bar indicates the number of nucleotide substitutions per site.

Within the eight genotypes of species *Orthohepevirus A,* the nomenclature of HEV subtypes is inconsistent, making the comparison of different studies on subtyping difficult. Recently, Smith et al. [64] proposed reference sequences for HEV subtypes within HEV-1 to HEV-7, including six subtypes (1a–1f) within HEV-1, two (2a and 2b) within HEV-2, 11 (3a–3j and 3ra) within HEV-3 and nine (4a–4i) within HEV-4. However, there are still huge numbers of unassigned subtypes, as shown in Figure 2a (unassigned subtypes marked with closed circles). Several research groups have proposed new subtype assignments according to the proposed criteria [64]. The most recent subtype assigned to the HEV-1 is subtype 1f [64]. Our lab recently proposed a new subtype, 1g (Figure 2a), consisting of HEV strains recovered from sporadic cases of imported (from Pakistan and India) and autochthonous acute hepatitis E in Japan [65].

HEV-3 strains are known to be highly divergent [66], and most unassigned subtypes belong to this genotype, as shown in Figure 2a. Miura et al. [67] proposed the new subtype 3k for four unassigned complete genomic sequences in Japan that were phylogenetically distinct from previously assigned complete genomic sequences, not related to any of the reported subtypes and epidemiologically unrelated (consisting of AB369689, AB740232 and LC176492 in Figure 2a). Another new subtype 3l was proposed in two different reports. The first was from Switzerland for HEV from a kidney transplant recipient [68], and the second was reported in swine strains from two farms in Northern Italy [69]. The HEV-3l strains reported by Wang et al. [68] differ from those reported by De Sabato et al. [69] by 13.3–14.4% over the entire genome, suggesting that they should be segregated into two distinct subtypes, according to the criteria proposed by Smith et al. [64]. An official assignment needs to be made by the International Committee on Taxonomy of Viruses (ICTV) to avoid confusion. In addition to these two newly proposed subtypes, there are still many unassigned strains within HEV-3 that can be defined as new subtypes 3m to 3s in HEVNet (https://www.rivm.nl/en/hevnet) as indicated in Figure 2a.

### 2.2. Orthohepevirus B

Isolates of *Orthohepevirus B* are restricted to birds, primarily chickens. This species is provisionally divided into four genotypes with different geographical distributions: genotype 1 is found in Australia, genotypes 2 and 3 in the USA and Europe and genotype 4 in Hungary [70,71,72,73]. However, the region-dependent genotype distribution does not apply for the avian HEV strains identified in China, Korea and Taiwan [74,75,76] (Figure 2b). This figure shows that the avian HEV strains from Korea are part of genotypes 1 and 2 of *Orthohepevirus B,* which was previously thought to only include avian strains from Australia and the USA; in contrast, the Chinese and Taiwanese avian strains are part of genotypes 3 and 4, respectively, which were previously thought to only include avian strains from Hungary. The members of *Orthohepevirus B* are associated with big liver and spleen disease and hepatitis-splenomegaly syndrome [77].

### 2.3. Orthohepevirus C

*Orthohepevirus C* is divided into HEV-C1, which has been isolated in several countries, including Germany, Australia, Belgium, Denmark, France, Italy, Spain, Switzerland, USA, Vietnam, Indonesia, China and Hong Kong (*Rattus spp.*, greater bandicoot rat, Asian musk shrew) [38,39,79,80,81,82,83,84,85,86,87], and HEV-C2 (ferret [88,89], mink [40]).

Although not assigned by the ICTV, recent reports from China and Brazil have described 12 novel rodent HEV strains representing at least nine clades of rodent HEVs in the *Orthohepevirus C* species [78,90,91] (Figure 2c, unassigned strains marked with closed circles), including putative HEV-C3 and HEV-C4 (Chevrier’s field mouse and Pere David’s vole) proposed by Wang et al. [78]. These 12 newly identified rodent HEV strains share only 49.5–65.4% nucleotide sequence identity over the entire genome with the prototype C1 rat HEV strain (GU345042), reinforcing the marked genomic variability among HEV strains. The kestrel-derived HEV strain was reported to form the same clade with the newly identified rodent HEV strains (Figure 2c), possibly due to its diet (e.g., voles, shrews and mice) [92]; however, this kestrel-derived HEV strain remains unclassified by the ICTV [23].

The zoonotic potential of HEV-C was previously suggested, as enzyme-linked immunosorbent assays (ELISAs) with virus-like protein indicated possible subclinical infection with HEV-C among forestry workers in Germany [93] and febrile inpatients in Vietnam [94]. Recently, HEV-C was demonstrated to cause infection in both an immunocompromised patient [95] and an immunocompetent patient [96].

### 2.4. Orthohepevirus D

*Orthohepevirus D* currently includes an isolate from a bat in Germany [97]. Although another bat HEV strain has been identified in China [98], it shares only 58% nucleotide sequence identity with the prototype bat HEV strain over the entire genome (Figure 1). Whether or not the Chinese bat HEV strain should be classified into the *Orthohepevirus D* species has not yet been determined by the ICTV. There is no evidence of transmission of bat HEV to humans.

### 2.5. Other Unassigned Related Hepeviruses

Several other related viruses are yet to be classified, including those from moose [99,100], fox [101], little egret [102], tree shrew (GenBank accession number KR905549) (Figure 1), sparrow [103] and agile frog [104].

## 3. HEV Genome

**Figure 3:**
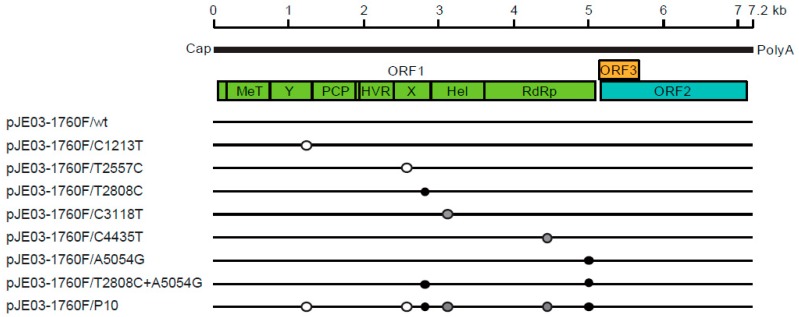
Positions of mutations in the eight recombinant cDNA clones of the JE03-1760F strain. Upper panel: the genomic structure of HEV. Abbreviations: MeT, methyltransferase; Y, Y domain; PCP, papain-like cysteine protease; HVR, hypervariable region; X, X domain; Hel, helicase; and RdRp, RNA-dependent RNA polymerase. Lower panel: the open circles denote synonymous mutations without structural changes in HEV RNA, the shaded circles denote synonymous mutations with structural changes in HEV RNA and the closed circles denote non-synonymous mutations. Modified from Nagashima et al. [187].

HEV is a single-stranded positive-sense RNA virus with a genomic length ranging from 6.6 to 7.2 kb (Figure 3). The genome contains a short 5’-untranslated region (5’-UTR) capped at the 5’-end, three open reading frames (ORFs) and a short 3’-UTR terminated by poly(A) tract [22,105]. ORF1 encodes the nonstructural proteins involved in viral replication [106]. ORF1 consists of seven functional domains, including methyltransferase domain (MeT), Y domain, papain-like cysteine protease (PCP), hypervariable region (HVR, or polyproline region), X domain (Macro domain), helicase domain and RNA-dependent RNA polymerase (RdRp). There have been conflicting reports regarding whether ORF1 products function as a single polyprotein or they need to be further processed into smaller units by viral or cellular proteases following translation [107,108]. ORF2 encodes the capsid protein [109], which plays a crucial role during virion assembly and viral attachment to the host cell and is the major target for neutralizing antibodies [110,111]. ORF3 is a small protein required for virion morphogenesis and virion egress [112,113,114]. Recently, ion channel activity and palmitoylation at cysteine residues, which are critical for the release of infectious particles, have been reported for the ORF3 protein [115,116]. ORF2 and ORF3 are translated from a single subgenomic RNA strand [117,118]. Members of the same genus all share similar genome organization. ORF3 overlaps with the 5’-end of ORF2 in *Orthohepevirus*, while in *Piscihepevirus*, the overlap is more central [119]. HEV is a quasi-enveloped virus in which the HEV particles in feces and bile are not enveloped, while those in the culture supernatant and circulating blood are covered by a cellular membrane and ORF3 protein [120,121,122,123]. ORF4 was identified in the coding sequence of ORF1 exclusively in HEV-1 and is said to play a role in increasing the RdRp activity [124]. ORF4 was also identified in rat HEV [79]; however, our recent study showed that putative ORF4 is not necessary for the active in vitro and in vivo replication of rat HEV [125].

## 4. Distribution and Clinical Significance of HEV Genotype within *Orthohepevirus A*

HEV infection is distributed around the world. HEV has been isolated in many countries in Asia, Europe, America, Africa and Oceania. HEV-1 is found in Asia and Africa, HEV-2 is found in Mexico and Africa, HEV-3 is found worldwide and HEV-4 is found mainly in Asia, including China, Hong Kong, India, Indonesia, Japan, Korea, Mongolia, Taiwan and Vietnam [126]. HEV-1 is also linked to imported infections in several European countries (i.e., Spain, Finland and France) [127] and industrialized Asian countries like Japan [65] that are isolated from patients with a history of traveling to endemic countries. HEV-1 and HEV-2 infections mostly affect developing countries. The infections by the two genotypes are responsible for the outbreaks in developing countries where the viruses are transmitted through drinking water supplies contaminated with human feces (e.g., after heavy rainfall or flood). The outbreaks were reported in refugee camps, military camps and internally displaced persons camps with dense populations and poor sanitation [2,3,4,7,9]. Since within the past decades HEV infection has been increasingly identified in developed countries, where the prevalent strains are HEV-3 and HEV-4, this section will focus mainly on these two genotypes. In Japan, subtypes 3b, 3a and 3e within HEV-3 and subtypes 4c and 4i within HEV-4 are predominant and indigenized, since they have been recovered from both hepatitis E patients and animals including domestic pigs and/or wild boars [128,129,130,131,132,133,134,135,136,137,138,139]. Although the global circulation of HEV-3 subtypes suggests that there are no clear regional demarcations, with subtypes no longer limited to their regions of origin [140], subtype 3b is indigenous to Japan, and no 3b strains have thus far been identified in other countries. Subtype 3l (see Figure 2a) is also reported to be indigenous to Switzerland [68].

Pigs are the primary reservoir of HEV. HEV has been isolated in pigs worldwide, but the infection is subclinical in this species [141]. A report from the Netherlands studying HEV transmission by contact-exposure in pig farms estimated that the basic reproduction rate (R_0_, number of individuals infected by an index case with an infectious disease) of HEV in a pig population is 8.8 (R_0_ > 1 means that the infection will spread through a naïve population), indicating that HEV is highly infectious in pigs. Once a pig in a herd becomes infected, it is extremely likely that all other animals in the herd will become infected as well [142]. The distribution of the subtypes can also be influenced by the import of animal products or live animals for breeding purposes, because they carry the risk of inflow and indigenization of foreign HEV strains [143,144].

Countries like China and Mongolia experienced a shift in the prevalent genotypes. In China, HEV-1 was previously the dominant circulating genotype; in recent years, however, HEV-1 has become less common, and HEV-4 is now the most prevalent genotype found in humans [145]. In Mongolia, the prevalent genotype has shifted from HEV-4 to HEV-1 [146]. In Japan, besides HEV-3, HEV-4, HEV-5 and HEV-6 isolated from humans, pigs, wild boars and deer, our recent study revealed the infection of HEV-1 in patients with no history of overseas travel that were subsequently regarded as autochthonous hepatitis [65]. In Southeast Asia, an increasing number of HEV strains have been isolated in the past few years. In Thailand, Cambodia and Singapore, HEV-3 was reported as the prevalent genotype in human populations, swine populations and blood products for transfusion [147,148,149,150,151,152]. HEV-4 was also isolated in Cambodia and Malaysia [153,154].

In European countries, HEV-3 is the prevalent genotype. However, HEV-4 has been increasingly frequently isolated in several European countries both from human and swine populations. The emerging autochthonous HEV-4 in Europe was probably transmitted by at least two distinct sources [155]. The first HEV-4 report came from a single autochthonous case in Germany (HEV-4f) [156] and swine in Belgium HEV-4b [157] in surveillance activities. France has the most HEV-4 cases reported in Europe. It was first isolated from a leukemia patient described as an autochthonous case [158], followed by two cases with HEV-4b linked to a history of figatelli consumption (pork liver sausage that is traditionally consumed uncooked) [159]. Several HEV-4b strains (including one case in a kidney transplant patient) were then isolated along with the imported Chinese HEV-4 strain [155,160,161]. An outbreak in Italy affecting five people living in the same area with no history of traveling to endemic areas was identified as HEV-4d, a strain that is close to Chinese swine isolates [162]. Denmark reported three HEV-4 cases—one that was close to the Italian outbreak strain, and two others that were close to the French figatelli cases and the Belgium swine strain [163]. HEV-4b was also isolated from a patient in Russia and thought to have been imported from France [164], and HEV-4 was isolated in the United Kingdom from an immunocompromised male patient (severe rheumatoid arthritis on treatment) with liver failure (jaundice and progressive encephalopathy) returning from India. The strain was close to the Indian HEV-4 isolated in swine [165]. A decade after the first report of HEV-4 in Europe, HEV-4b has been proven the most prevalent subtype among HEV-4 infections reported from Europe.

HEV-1 is known to be related to the development of fulminant hepatitis in pregnant women in Asia and Africa [9,166]. However, several reports have described the rare involvement of HEV-3 in pregnant women from areas such as Germany and France (HEV-3c), southeastern France (HEV-3f) and Japan (HEV-3b) [167,168,169,170].

In industrialized countries, HEV genotypes in hepatitis patients are generally the same as those in swine populations, suggesting zoonotic transmission by food or close contact with animals. In contrast, in developing countries, infection can be epidemic or sporadic, and the strains found in humans tend to differ from those isolated from pigs [171].

Animal strains of HEV are being isolated increasingly frequently worldwide. Several new strains were only able to be discovered in restricted locations due to limited screening (e.g., common kestrel, red-footed falcon and little egret in Hungary) [92,102]. Expansion of screening areas and animal species might uncover even more new animal strains.

Despite their similar modes of transmission and ability to cause chronic hepatitis in immunosuppressed patients, several reports have demonstrated differences in the clinical features and pathogenesis of HEV-3 and HEV-4. Several studies from Japan have shown that HEV-4 patients had a significantly higher peak alanine aminotransferase (ALT) level and significantly higher proportion of prothrombin time (PT) ≤60% than HEV-3 patients, and that fulminant hepatitis events were significantly more frequent in HEV-4 patients than in HEV-3 patients, suggesting that the HEV genotype is an important risk factor associated with the disease severity [136,172,173]. These findings in Japan were also observed by a study in France. The authors found that patients infected with HEV-4 showed significantly higher ALT levels and more frequent jaundice events than those with HEV-3 infection [160]. The first isolation of HEV-4 in swine in Belgium was followed by experimental infection in swine to test the infectivity of the HEV-4 isolate [157]. The ALT and aspartate amino transferase (AST) levels in pigs experimentally infected with HEV-4 isolate were higher than those observed in pigs experimentally infected with HEV-3. This observation suggests that HEV-4 may cause more severe liver damage than HEV-3 [157].

Reports on HEV-4 infection in immunocompromised patients (cancer patients and transplant recipients) demonstrated that six out of seven cases progressed to persistent infection. In three cases, infection did not respond to ribavirin or relapsed despite the administration of ribavirin [174,175,176,177,178]. Combined data from two studies in the same center showed that 89% of the HEV-4-infected transplant recipients developed persistent infection. In the same studies, six patients received a reduced dose of immunosuppression, but with no effect on their viral load [176,179]. This observation differed from what was demonstrated in chronic HEV-3 cases where infection was cleared spontaneously in 34%, while the infection cleared in 21% after the reduction of immunosuppression [180]. An analysis of the RdRp sequence in serial specimens (including the baseline) of immunosuppressed patients with chronic HEV-4 infection who responded poorly to ribavirin treatment showed that the K1383N mutant found after nine months of ribavirin treatment remained at 15 and 18 months of ribavirin treatment, a finding similar to that reported in HEV-3 [176]. In contrast with this finding, an in vitro experiment showed that this mutation leads to increased susceptibility to ribavirin and reduced viral fitness of HEV-3 [181]. In HEV-3, aa 1634 (the RdRp region of ORF1) is glycine [181], while it was lysine in HEV-4 that was conserved during ribavirin treatment [176]. In ribavirin-resistant HEV-3 mutants, this amino acid frequently exhibited a glycine-to-arginine mutation [181]. The findings of mutational impact in the outcomes of patients treated with ribavirin warrant further studies.

Both HEV-3 and HEV-4 infections in immunocompromised patients have been linked to accelerated cirrhosis; however, a small number of reports have shown earlier progression to cirrhosis in those with HEV-4 infection than in those with HEV-3 infection [175,179,182]. A report of two patients with chronic HEV-3 infection demonstrated the development of liver cirrhosis within less than three years [182], while other small reports showed that two liver graft recipients experienced the rapid development of cirrhosis in a matter of months (less than one year) after being diagnosed with HEV-4 infection, both of whom died due to complication of esophageal variceal bleeding [175,179]. A comparative study of HEV-3- and HEV-4-infected patients in terms of accelerated liver cirrhosis compared with the non-HEV-infected liver cirrhosis patients is necessary to further confirm this finding. Although a recent systematic review and pooled analysis on acute liver failure (ALF) caused by HEV-3 and HEV-4 suggested that there were no major differences between patients infected with HEV-3 versus HEV-4 [183], the observed differences in the clinical features, pathogenesis and prognosis of HEV-3 and HEV-4 infection merit a further analysis.

## 5. Genomic Variability and Evolution

HEV strains have demonstrated extensive genomic diversity among them. Although HEV strains are highly diverse and heterogeneous, only one serotype of HEV exists. This is probably related to the high degree of conservation of the amino acid sequence of the capsid protein among distinct genotypes, correlating with the little antigenic diversity [66]. HEV genotypes have diverse reservoirs, distinct distribution and varied transmission pattern. This variability contributes to the pathophysiology, transmission patterns, severity of the infection, and probably to therapeutic response as well [184,185].

### 5.1. Nucleotide Mutations during Consecutive Passages in Cell Culture (Clinical Sample-Derived versus cDNA Clone-Derived)

Cell culture-derived adaptive mutations can greatly improve the in vitro replication capacity of the virus, as has been demonstrated by studies in our lab using the HEV-3 JE03-1760F strain. Adaptation to growth in cell culture reduces the interval between inoculation of cultures and maximizes the viral yield. Mutations can occur frequently over the entire HEV genome during propagation and consecutive passages for adaptation to cell culture [185,186,187]. Random mutations might occur during passages for adaptation to growth in cell culture. The mutations important for the virus can in part be suggested by the reproducible occurrences observed in independent experiments using the same inoculum.

Previously, our lab performed consecutive passages of two starting viruses―the feces-derived JE03-1760F/wild type (wt) (experiment A and experiment B) and the infectious cDNA clone-derived pJE03-1760F/wt―to characterize genomic mutations of HEV during consecutive passages associated with adaptation to growth in cell culture. During the passages, increased growth efficiency was observed in both feces-derived and infectious cDNA clone-derived viruses. To determine the molecular mechanism underlying the adaptation of JE03-1760 to growth in cell culture, full-genome sequencing and a comparison with the wild-type parent were performed [185,187]. The full genome sequences of passage 10 (feces-derived p10f/A, feces-derived p10f/B and cDNA-derived p10c) are presented in Table 2.

In feces-derived passages (experiment A), the average time required by p0–p5 to reach an HEV RNA titer of 1 × 10^5^ copies/mL was 35.2 days, while it was 16.0 days for p6–p10, which means that p6–p10 reached the target titer 19.2 days earlier. A direct comparison showed that it took 40 days for p1 to reach the titer, while it took 12 days (28 days earlier) for p10 to do so. However, experiment B, which used the same inoculum as experiment A, found that the average time required by p6–p10 to reach 1 × 10^5^ copies/mL was one week less than the time for p0–p5 to reach the same titer. Full-genome sequencing revealed that, in experiment A, the total number of mutations accumulated over 10 consecutive passages was 18 (18/7226 or 0.25%), with five amino acid substitutions, while this value was 22 (22/7226 or 0.30%), with nine amino acid substitutions, in experiment B [185,186]. The limited number of mutations found in these experiments was also observed in passages of another enterically transmitted hepatitis virus, hepatitis A virus (HAV), where HAV variants of passage 16 HM175 (16th in vitro passage level) exhibited 19 mutations accounting for 0.3% of the entire genome of the parent virus [188].

In infectious cDNA clone-derived passages, the average time required by p1–p5 to reach an HEV RNA titer of 1 × 10^5^ copies/mL was 17.0 days, while it was 7.8 days for p6–p10, which means that p6–p10 reached this titer 9.2 days earlier. A direct comparison showed that it took 31 days for p1 to reach the titer, while it took only five days (26 days earlier) for p10 to do so. Full-genome sequencing revealed that the total number of nucleotide mutations accumulated over 10 consecutive passages was six (6/7226 or 0.08%), with two amino acid substitutions. Eight new infectious cDNA clones based on these six mutations (six individual nucleotide mutations (C1213T, T2557C, T2808C, C3118T, C4435T, A5054G); two amino acids substitution (T2808C + A5054G); and all six nucleotide mutations) were then constructed (Figure 3) in order to confirm the results. Compared to the wild-type virus, T2808C + A5054G showed a higher viral load (10-fold), while the infectious cDNA clone with all six mutations demonstrated a 100-fold-higher viral load than the wild-type virus. This result suggests that the virus is adapted to growth in cell culture. Among the clones with four individual mutations, two individual mutants (C1213T and T2557C) with no amino acid substitutions demonstrated faster viral growth than the wild-type virus. In these two mutants, minimal changes in the secondary structure of the RNA sequence were observed (Figure 3). In contrast, the other two individual mutants (C3118T and C4435T) showed similar growth to the wild-type virus. In those two mutants, the secondary structures were not changed, suggesting that changes in the secondary structures might affect the viral replication capacity [187].

A common mutation (T2808C) found in experiment B and the infectious cDNA clone experiment plays an important role in heightened virus replication, as shown by the results indicated above; therefore, the common mutations found in experiments A and B (three) might play important roles in heightened virus replication as well. The finding of common mutations in the two independent experiments (A and B) suggests the possible role they may play in heightened virus replication, which was further proven by the reproducible occurrence observed in two independent experiments. The extended in vitro passage of the virus may result in the virus attenuation, an approach that could be utilized for the development of attenuated HEV vaccine in the future.

### 5.2. Possible Clinical Implication of HEV Genomic Mutations

HEV infections in humans have been caused by five different genotypes whose genomic organization is highly conserved [189]. The HEV strains capable of infecting humans were previously thought to be restricted to HEV-1, HEV-2, HEV-3 and HEV-4. However, the range expanded following reports of human infection through the regular consumption of camel meat and milk in the United Arab Emirates (HEV-7) [60]. HEV-1, HEV-2, HEV-3, HEV-4 and HEV-7 are all classified under the genus *Orthohepevirus A*. The host range has been found to expand to another species—*Orthohepevirus C*—as the first case of HEV-C1 (rat) infection in an immunocompromised patient (a recipient of liver transplant) was reported from Hong Kong [95]. Within one month, another case of HEV-C1 infection in a human was reported from an immunocompetent Canadian male [96]. However, how the rat HEV was transmitted to humans remains unclear at present. The emergence of rat HEV infecting both immunocompromised and immunocompetent humans raises the possibility that rat HEV strains infecting humans in wider geographic areas around the world might be discovered in the future, as rat HEV strains have been isolated increasingly frequently in many parts of the world, including several European countries [79,86], the US [80] and Asian countries [38,83,84,85,87].

Infection with HEV-1 and HEV-2 is restricted to humans, while HEV-3 and HEV-4 have a broader host range, including humans as well as a variety of animals, such as pigs, wild boars, rabbits, mongoose and deer. HEV-1 is the most conserved among the main four genotypes, while HEV-3 and HEV-4 strains are highly diverse. HEV host specificity is a heritable and convergent phenotypic trait that can be achieved independently by various HEV-3 and HEV-4 strains through many genetic pathways, explaining the broad host range for HEV-3 and HEV-4 [190]. Amino acid positions 605, 1017 and 1252 in helicase, which have been associated with severe hepatitis in HEV-3-infected patients [191], were found to be some of the most influential sites of the HEV-3 ORF1-encoded protein. All three sites were involved in a Bayesian Network (BN)_HEV3_. In addition, position 1252 was recognized as an HEV-3 host-specific motif, with position 605 being a part of the human motif. These observations suggest the possibility that the host-specific coevolution among protein sites is associated with HEV virulence [190].

HEV-1 is linked to severe forms of liver disease and complications in pregnant women. A recent work in North India reported molecular alterations in the partial sequence of the RdRp region from patients with acute liver failure (ALF) and acute viral hepatitis (AVH), including pregnant women, and its association with the poor outcome of the disease. They demonstrated two novel mutations—Cysteine 1483 Tryptophan (C1483W) and Asparagine 1530 Threonine (N1530T)—in 100% (25/25) of the patients with ALF compared to none (0/30) of the patients with AVH (*p* < 0.0001). The disease severity parameters and viral load in samples with C1483W and N1530T mutations were significantly higher than in those lacking the mutation. This means that the mutations are associated with the outcome in ALF patients. The nucleotide substitutions in the RdRp region may play an important role in enhancing HEV replication, thereby leading to disease severity [192].

HEV-1 and HEV-2 are associated with acute infection, while HEV-3, HEV-4 and HEV-7 not only cause acute hepatitis, but can lead to chronic infection in immunocompromised patients. A small study involving 14 solid organ transplant recipients in France [184] demonstrated that the complexity and diversity of the polyproline region (PPR) and macro domain in ORF1 were higher in patients whose HEV infection became chronic compared with those who cleared the virus, suggesting a great quasispecies heterogeneity in these regions [184]. As the PPR could modulate the host immune response, and the macro domain could influence virus pathogenicity [184,193,194,195], the genetic heterogeneity of the PPR and the macro domain may play a role in the outcome of HEV infection in immunocompromised patients (e.g., the solid organ transplant recipients) that could facilitate HEV persistence [184,196].

The high variability and frequent selection of mutations in the HEV genome are due to the transcription process [197]. Mutations can occur frequently over the entire HEV genome during propagation and consecutive passages for adaptation to cell culture [185]. The HEV mutation rates were estimated indirectly from clinical isolates as 1.5 base substitutions per site per year [43]. Selection pressure imposed by antiviral drugs and host immune responses may contribute to increased HEV variability [184]. Non-synonymous substitutions can modulate viral proteins structurally and thus dysregulate virus-host interactions [197].

Several reports have described the HEV nucleotide mutations related to ribavirin treatment. The virus can acquire mutations that make intra-host populations less sensitive or even resistant to ribavirin. One of the proposed modes of action is a direct mutagenic effect on viral genomes, inducing mismatches and subsequent nucleotide substitutions [198]. Ribavirin resistance was associated with Y1320H, K1383N and G1634R mutations in the viral polymerase, along with an insertion in the HVR comprising a duplication and a polymerase-derived fragment. Mutations Y1320H and G1634R and the HVR insertion compensated for K1383N-associated replication defects [181]. A recent report from Singapore also described mutational hotspots within ORF3 and the PCP/HVR domain of ORF1 [152]. The viral heterogeneity related to ribavirin treatment was reversible when treatment was stopped [199].

HEV has been constantly evolving in order to adapt to new hosts. Most of the HEV genome is evolutionarily constrained. HEV-1, which infects humans only, has been evolving differently from HEV-3 and HEV-4, which infect multiple species. This is probably because HEV-3 and HEV-4 are unable to achieve the same fitness due to repeated host jumps [171]. HEV-3 and HEV-4 are enzootic and zoonotic, and capable of infecting a number of different species. The adaptation of each strain to a range of hosts may lead to a greater demand for genetic changes in the genome [200]. A greater number of polymorphic positions were carried by HEV-3 and HEV-4 compared with those of HEV-1 and HEV-2, suggesting a high genetic diversity of HEV-3 and HEV-4 that may reflect their strong adaptation to many hosts. Under different selective pressures, many positive selection (mutations leading to amino acid substitution) sites were located in the overlapping region of ORF2 and ORF3. Meanwhile, the ORF1 and the non-overlapping ORF2 have many negative selections sites (silent mutations) that were greater in HEV-1 compared with those in HEV-3 and HEV-4, which may explain why HEV-1 is well conserved and adapted only by human hosts [200,201]. A divergence analysis of HEV-1 to HEV-4 suggested that the split into zoonotic and anthropotropic genotypes occurred around 536 to 1344 years ago. HEV-1 appears to be more recent than the zoonotic genotypes, with the estimated time to the most recent common ancestor (tMRCA) of most modern lineages of HEV-1 being roughly ~87 to 199 years ago. The population dynamics of HEV-1, HEV-3 and HEV-4 over the last century have demonstrated the association of effective population size with global trade, wars, fluctuations in pork consumption and the increased recognition of hepatitis E as a result of zoonosis and control measures in swine [200,202].

## 6. Conclusions

Over the past two decades, HEV strains have been isolated with increasing frequency not only from humans but also from other animal species, necessitating revisions to the previous consensus, as many of the newly identified strains remain unclassified. It is possible that more strains will be discovered from even more diverse animal species in the future. This variability among HEV genotypes contributes to the pathophysiology, transmission patterns, severity of the infection and likely therapeutic response. Evolutionary events have conferred the ability of HEV to adapt to new hosts.

## Figures and Tables

**Table 1:** Classification of hepeviruses.

Family	Genus	Species	Genotype	Host
*Hepeviridae*	*Orthohepevirus*	*Orthohepevirus A*	1	human
			2	human
			3	human, pig, wild boar, deer, mongoose, rabbit, goat ^a^, horse ^b^, bottlenose dolphin ^c^, sheep ^d^
			4	human, pig, wild boar, cattle ^e^, cow ^f^, sheep ^g^, goat ^h^, yak ^i^
			5	wild boar
			6	wild boar
			7	dromedary camel
			8	Bactrian camel
		*Orthohepevirus B*		chicken
		*Orthohepevirus C*	C1	rat, greater bandicoot rat ^j^, Asian musk shrew ^k^
			C2	ferret, mink ^l^
		*Orthohepevirus D*		bat
	*Piscihepevirus*	*Piscihepevirus A*		cutthroat trout

^a^ Di Martino et al. [25]; ^b^ Saad et al. [26]; Zhang et al. [27]; Geng et al. [28]; Garcia-Bocanegra et al. [29]; ^c^ Montalvo Villalba et al. [30]; ^d^ Sarchese et al. [31]; ^e^ Hu and Ma [32]; ^f^ Vitral et al. [33]; Huang et al. [34]; ^g^ Wu et al. [35]; ^h^ Li et al. [36]; ^i^ Xu et al. [37]; ^j^ Li, W. et al. [38]; ^k^ Guan et al. [39]; ^l^ Krog et al. [40].

**Table 2:** A comparison of sequences of the wild-type (wt) JE03-1760F, its cell culture-produced variants (feces-derived) and the cell culture-produced variant of pJE03-1760F/wt (cDNA-derived) over the entire genome.

Nucleotide Position	Region	Nucleotide				Amino Acid	
		JE03-1760F/wt	p10f/A (Feces-Derived)	p10f/B (Feces-Derived)	p10c (cDNA-Derived)	Position	Substitution
22	5’UTR	U	A	U	U	NA ^a^	-
61	ORF1 (MeT)	U	U	C	U	12	-
370	ORF1 (MeT)	C	U	C	C	115	-
445	ORF1 (MeT)	U	U	C	U	140	-
591	ORF1 (MeT)	C	U	C	C	189	Ala to Val
829	ORF1 (Y)	C	C	U	C	268	-
1213	ORF1 (Y)	C	C	C	U	396	-
1378	ORF1 (PCP)	C	C	U	C	451	-
1549	ORF1 (PCP)	U	U	C	U	508	-
2191	ORF1 (HVR)	C	C	U	C	722	-
2236	ORF1 (HVR)	C	C	U	C	737	-
2246	ORF1 (HVR)	U	**C**	**C**	U	741	Trp to Arg
2557	ORF1 (X)	U	U	U	C	844	-
2704	ORF1 (X)	U	C	U	U	893	-
2808	ORF1 (X)	U	U	**C**	**C**	928	Val to Ala
2913	ORF1 (Hel)	A	A	G	A	963	Glu to Gly
2915	ORF1(Hel)	G	G	U	G	964	Val to Leu
2938	ORF1 (Hel)	C	U	C	C	971	-
3106	ORF1 (Hel)	A	G	A	A	1027	-
3118	ORF1 (Hel)	C	C	C	U	1031	-
3223	ORF1 (Hel)	U	U	C	U	1066	-
3235	ORF1 (Hel)	C	U	C	C	1070	-
3453	ORF1 (Hel)	C	U	C	C	1143	Ala to Val
3475	ORF1 (Hel)	C	C	U	C	1150	-
3496	ORF1 (Hel)	C	U	C	C	1157	-
4015	ORF1 (RdRp)	C	U	C	C	1330	-
4309	ORF1 (RdRp)	C	C	U	C	1428	-
4435	ORF1 (RdRp)	C	C	C	U	1470	-
4462	ORF1 (RdRp)	C	U	C	C	1479	-
5054	ORF1 (RdRp)	A	A	A	G	1677	Ile to Val
5312	ORF2	U	U	C	U	47	-
	ORF3					51	Ile to Thr
5378	ORF2	A	**G**	**G**	A	69	-
	ORF3					73	Asn to Ser
5456	ORF2	C	**U**	**U**	C	95	-
	ORF3					99	Pro to Leu
6047	ORF2	U	U	C	U	292	-
6470	ORF2	C	U	C	C	433	-
6578	ORF2	C	U	C	C	469	-
6611	ORF2	C	U	C	C	480	-
6626	ORF2	U	C	U	U	485	-
6652	ORF2	U	U	C	U	494	Val to Ala
6855	ORF2	A	A	G	A	562	Asn to Asp
6944	ORF2	U	U	C	U	591	-
7186	3’UTR	C	C	U	C	NA ^a^	-

Made from Lorenzo et al. [185], Okamoto [186] and Nagashima et al. [187]. ^a^ Not available. MeT: methyltransferase; Y: Y domain; PCP: papain-like cysteine protease; HVR: hypervariable region; X: X domain; Hel: helicase; RdRp: RNA-dependent RNA-polymerase. Green background color indicates nucleotide mutation.
